# Conversion to Curative Resection and Pathological Complete Response Following Targeted Therapies With Atezolizumab and Bevacizumab for Initially Unresectable Hepatocellular Carcinoma: A Case Report

**DOI:** 10.7759/cureus.45176

**Published:** 2023-09-13

**Authors:** Taisuke Matsuoka, Takahisa Fujikawa, Yusuke Uemoto, Yuki Aibe, Suguru Hasegawa

**Affiliations:** 1 Surgery, Kokura Memorial Hospital, Kitakyushu, JPN; 2 Gastroenterology, Kokura Memorial Hospital, Kitakyushu, JPN; 3 Gastroenterological Surgery, Fukuoka University Hospital, Fukuoka, JPN

**Keywords:** robotic liver resection, conversion surgery, pathological complete response, atezolizumab plus bevacizumab, hepatocellular carcinoma

## Abstract

Hepatocellular carcinoma is a malignancy with an increasing incidence worldwide and is one of the most serious cancers in adults. We encountered a case of initially unresectable massive hepatocellular carcinoma in which conversion to curative resection and pathological complete response were achieved after atezolizumab plus bevacizumab therapy. Atezolizumab plus bevacizumab combination chemotherapy may be one of the most promising options for unresectable hepatocellular carcinoma.

## Introduction

The incidence of hepatocellular carcinoma (HCC), the sixth most frequent cancer worldwide, is rising. The best curative choices for locoregional HCC remain resection and transplant. Regarding advanced HCC, new advancements in therapies such as programmed death-ligand 1 (PD-L1) inhibitors and antibodies for the vascular endothelial growth factor receptor (VEGF) have altered management [1].

The combination of the PD-L1 monoclonal antibody atezolizumab and the anti-VEGF monoclonal antibody bevacizumab became the first-line treatment for unresectable HCC [2]. Furthermore, after receiving atezolizumab plus bevacizumab therapy, there have been some instances of conversion surgery for unresectable HCC [3,4].

Regarding surgery, robotic liver resections have broadened their indications and gained acceptance lately [5,6]. When compared to open operations, these can be safely performed with equivalent oncological outcomes while causing less blood loss, a speedier recovery from surgery, and less postoperative pain [7,8]. We present a case of initially unresectable massive hepatocellular carcinoma in which conversion to curative robotic liver resection and pathological complete response were achieved after atezolizumab plus bevacizumab therapy.

## Case presentation

**Figure 1:**
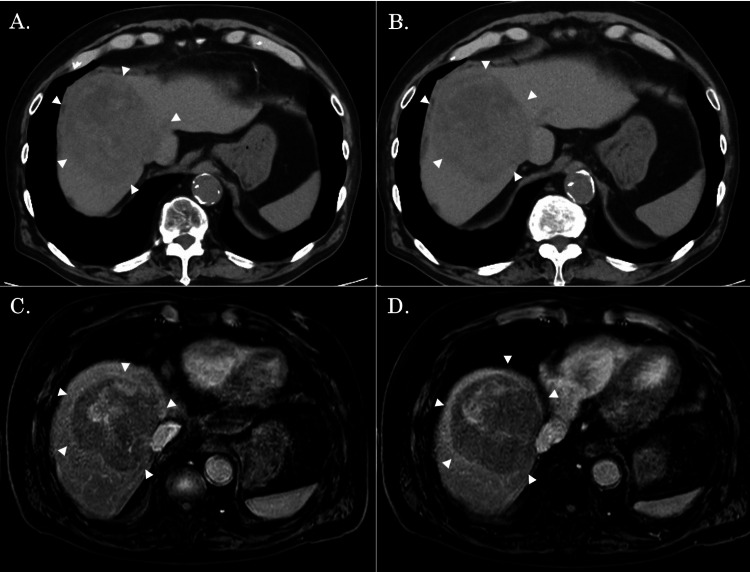
CT and MRI findings before the chemotherapy. (A, B) An enhanced CT scan showed that the tumor occupied a large space in the right lobe of the liver and was 82 × 78 mm in size (white arrows). (C, D) An MRI showed that the tumor was in contact with the roots of the inferior vena cava and the right and middle hepatic veins (white arrows).

An 85-year-old man was referred to our hospital for treatment of HCC. He had chronic liver disease due to hepatitis B virus and hepatitis C virus infections. An enhanced computed tomography (CT) scan showed a liver tumor at 82 × 78 mm in the right hepatic lobe in broad contact with the inferior vena cava and the root of the right and middle hepatic veins (Figure 1). Initial tumor marker levels were as follows: alpha-fetoprotein (AFP), 1,439 ng/mL; and protein induced by vitamin K absence or antagonist-II (PIVKA-II), 5,371 mAU/mL.

**Figure 2:**
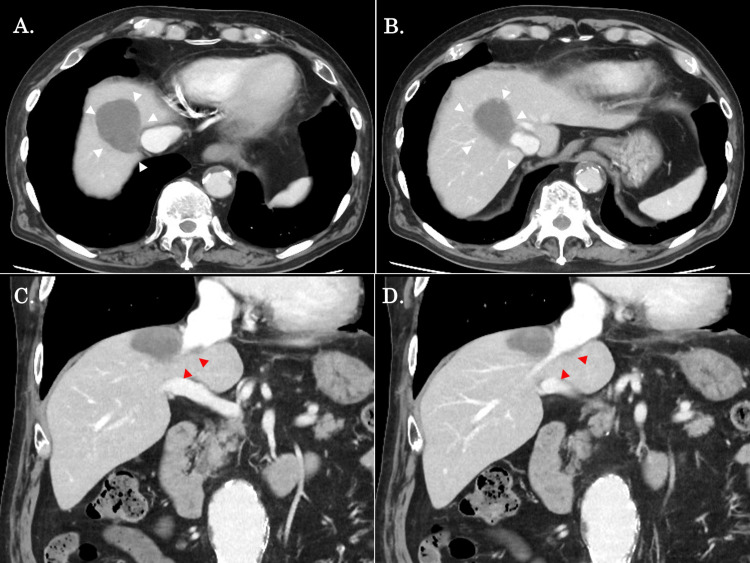
Contrast-enhanced CT findings after the chemotherapy. (A, B) An axial contrast-enhanced CT scan revealed a reduction in tumor size of 48 × 39 mm (white arrows). (C, D) Coronal sections showed that the tumor was still in contact with the right hepatic vein, but the proximity to the inferior vena cava and middle hepatic veins was alleviated (red arrows).

We considered the current tumor unsuitable for resection due to its size and location and immediately introduced the patient to combination biological therapy with atezolizumab (1,200 mg) plus bevacizumab (10 mg/kg). After the second course of biological therapy, his tumor marker levels were within the normal range (AFP: 2.3 ng/mL and PIVKA-II: 19.7 mAU/mL). Courses one to five were managed with atezolizumab plus bevacizumab, and atezolizumab monotherapy without bevacizumab was administered during the following four courses due to proteinuria. His tumor marker levels after the ninth course remained within the normal range (AFP: 1.8 ng/mL and PIVKA-II: 28 mAU/mL). A follow-up CT scan showed a remarkable reduction of the tumor size to 48 × 39 mm after the ninth course (Figure 2).

**Figure 3:**
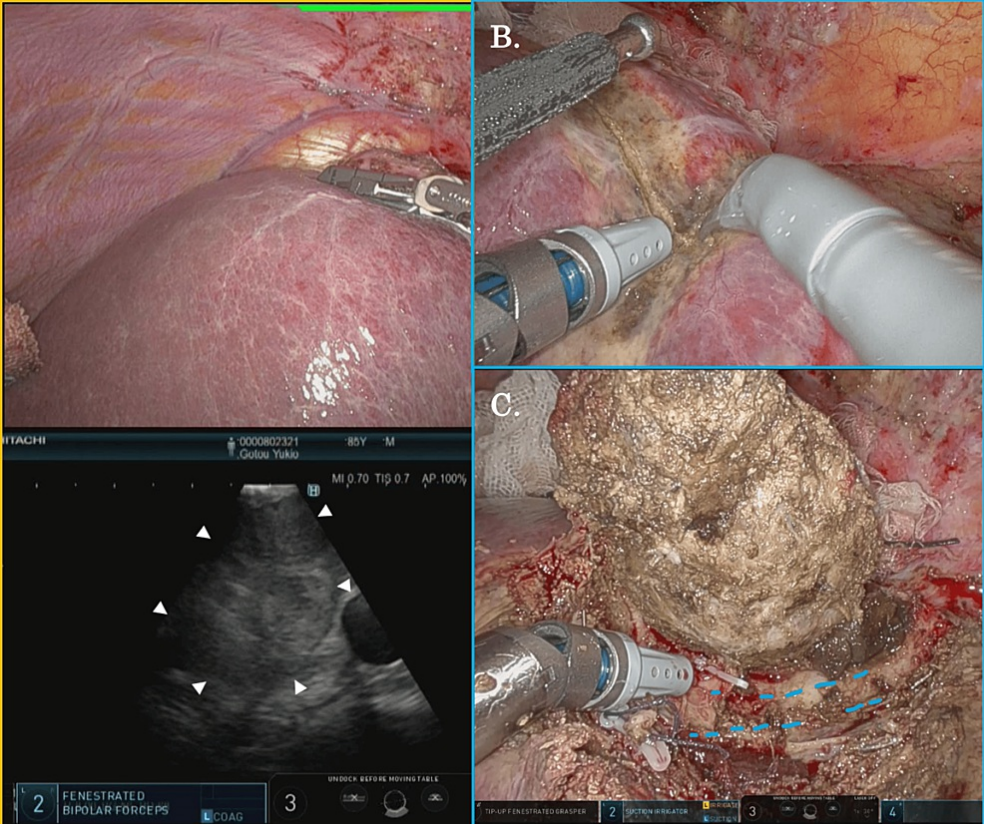
Operative findings during robotic S8vent anatomical liver resection. (A) An intraoperative ultrasound identified a tumor in liver S8 (arrows indicate the tumor). (B) Liver parenchymal transection using saline-linked monopolar scissors was performed during robotic liver resection. (C) Hepatectomy was completed while preserving both the middle and right hepatic veins (blue dotted lines).

At that point, liver resection with the preservation of major hepatic veins was considered possible, and surgery was planned. Preoperative liver function was graded as Child-Pugh class B (7 points). CT volumetry showed a liver volume of 890 mL and a residual liver volume of 846 mL in the case of anatomical resection of the S8 ventral area. During the operation, we found that the clearly demarcated hepatic tumor was located adjacent to both the middle and right hepatic veins but was able to be detached from them relatively easily. Using the saline-linked monopolar scissors (SLiC-Scissors) method, a previously reported novel technique for robotic liver parenchymal transection [9], robotic S8vent anatomical liver resection was completed without major incidents (Figure 3).

**Figure 4:**
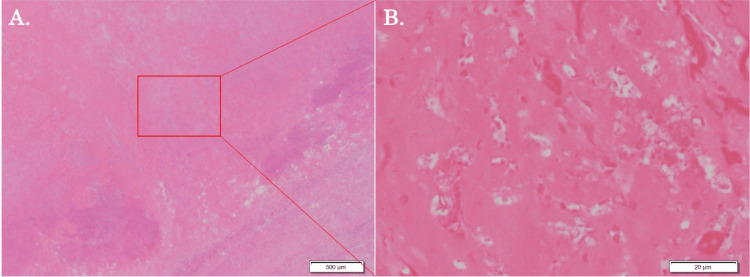
Pathological findings. Hematoxylin and eosin staining of the tissues revealed no viable cancer cells. (A) A low-power-field view of the lesion. (B) A high-power-field view of the lesion.

The postoperative pathological examination revealed R0 resection and pathological complete response (Figure 4). The recovery went without any incident, and he was discharged home on postoperative day nine. The patient is still doing well and free from oncological therapy without any recurrence eight months after the operations (14 months after the initial diagnosis).

## Discussion

Patients with localized HCC may be cured by surgery, ablation, or liver transplantation. On the other hand, the prognosis for HCC with metastases and locally advanced HCC was four to seven months before the invention of sorafenib [10]. As a multi-kinase inhibitor, sorafenib predominantly inhibits RAF kinase and VEGF receptor 2 (VEGF-R2) [11]. Since then, molecularly targeted drugs and PD-L1 monoclonal antibodies have taken over as the main therapies for advanced HCC. Atezolizumab plus bevacizumab combination chemotherapy showed good anti-tumor activity in patients with previously unresectable HCC, with a median progression-free survival of 5.6 months, an overall response rate of 27.3%, and a complete response of 5.5% in a Phase 1b study [2,12]. In addition, certain cases of conversion surgery for unresectable hepatocellular carcinoma following atezolizumab and bevacizumab therapy have been reported [3,4].

In our case, an enhanced CT scan at the time of the first visit showed extensive contact with the inferior vena cava and the right and middle hepatic veins, and curative-intent resection required the extended right lobectomy with combined resection of the middle hepatic vein; therefore, we thought upfront surgical resection was not suitable. Because it is strongly recommended by the 2021 Japanese Guidelines on Liver Cancer Examination and Treatment that atezolizumab plus bevacizumab combination therapy should be used as the first-line therapy for unresectable HCC due to its effect of extended survival more than sorafenib [2], we decided to select this regimen for this patient. Our treatment strategy was this combination therapy first, and if a tumor decrease was seen, we made the decision to perform a liver resection. Atezolizumab and bevacizumab combination therapy was extraordinarily successful. AFP and PIVKA-II returned to normal at the conclusion of the second treatment. The tumor size had also decreased by the conclusion of the ninth course, making it possible to separate the tumor from the right and middle hepatic veins. During the robotic anatomical liver resection, we found that the right and middle hepatic veins, as well as the inferior vena cava, were not involved by the tumor and were fully preserved. Moreover, pathological examination showed no residual tumor, and a pathologically complete response was achieved.

Regarding surgery, since the first laparoscopic liver resection was reported in 1991, it has spread rapidly worldwide. Oncological outcomes from minimally invasive liver surgery have been reported to be equivalent to those from open surgery. Minimally invasive surgery has advantages over open surgery for postoperative pain, intraoperative blood loss, postoperative complication rate, length of hospital stays, and quality of life [13]. Concerning robotic liver resection, it was initially documented by Giulianotti et al. in 2003 and has been reported frequently since then [14]. Robotic-assisted surgery has several advantages over conventional laparoscopy. It provides a magnified view of small structures, a three-dimensional surgical view, suppression of handshaking, instrument flexibility, and minimally invasive surgical approaches.

In this case, we were able to safely dissect the tumor from the right and middle hepatic veins, taking advantage of the superiority of robotic surgery. Thus, robotic liver resection after intensive biological therapy using atezolizumab and bevacizumab may be one of the promising options for unresectable HCC. However, a large number of HCC patients will be required to investigate the characteristics of cases in which atezolizumab plus bevacizumab combination chemotherapy is remarkably effective because this is a single case report.

## Conclusions

We report our experience with the robotic S8vent anatomical liver resection after atezolizumab plus bevacizumab combination therapy for initially unresectable HCC. The postoperative pathological examination revealed that a pathologically complete response was achieved by biological therapy, and the patient is doing well and free from oncological therapy. Surgical resection after atezolizumab plus bevacizumab combination therapy may be one of the most promising options for the treatment of unresectable HCC.
